# Total uterine prolapse complicated with vesicovaginal fistula: A case report

**DOI:** 10.1097/MD.0000000000026386

**Published:** 2021-06-18

**Authors:** Ning-Shiuan Ting, Hsiang-Chen Lee, Jia-Ying Ke, Pei-Chen Li, Dah-Ching Ding

**Affiliations:** a Department of Obstetrics and Gynecology, Hualien Tzu Chi Hospital, Buddhist Tzu Chi Medical Foundation; b School of Medicine, College of Medicine; c Institute of Medical Sciences, Tzu Chi University, Hualien, Taiwan.

**Keywords:** hysterectomy, pelvic organ prolapse, sacrospinous ligament fixation, vesicovaginal fistula

## Abstract

**Rationale:**

Vesicovaginal fistula (VVF) most commonly occurs due to iatrogenic injury during surgery or obstructed labor. We report a rare case of a patient with severe pelvic organ prolapse who developed VVF even though pessary had not been used.

**Patient concerns:**

A 63-year-old postmenopausal woman, para 3 (all spontaneous vaginal deliveries), complained of vaginal bulging sensation and involuntary urinary leakage for 3 years.

**Diagnosis:**

Stage IV uterine prolapse with VVF.

**Interventions:**

She underwent transvaginal VVF repair combined with total vaginal hysterectomy and sacrospinous ligament fixation. The postoperative course was uncomplicated.

**Outcomes:**

The patient remained free of complications during the 1-year follow-up.

**Lessons:**

This case illustrates the point that patients with pelvic organ prolapse (POP) should be treated promptly and careful follow-up should be conducted. Clinicians should be aware of the symptoms of VVF to ensure its early diagnosis and treatment.

## 1 Introduction

Vesicovaginal fistula (VVF) is an abnormal connection between the bladder and the vagina, which causes involuntary continuous urinary leakage from the vagina. In developed countries, the most common etiology of VVF is gynecological surgery, especially hysterectomy. A study conducted in the United Kingdom showed that the overall rate of urogenital fistula was 0.012% after all types of hysterectomies with the highest incidence occurring following radical hysterectomy for cervical cancer (1.14%) and lowest following vaginal hysterectomy for prolapse (0.02%).^[1]^ In developing countries, VVF is usually caused by prolonged obstructed labor leading to pressure necrosis which occurs in 2% of the patients with obstructed labor. Poor socioeconomic status, early marriage, malnourishment, low literacy rate, and poor health-care system contribute to the higher prevalence of VVF in these countries.^[2]^

Pelvic organ prolapse (POP) refers to a falling, slipping, or downward displacement of the uterus and/or different vaginal compartments and their neighboring organs such as the bladder, rectum or bowel.^[3]^ General symptoms of POP include pressure, heaviness, presence of a bulge, or sensation of “something falling out of the vagina.” Additionally, specific symptoms related to the site of the lesion may also be present. Women with anterior vaginal prolapse (cystocele) may need to digitally reduce their prolapse (urinary splinting) to aid with voiding while those with posterior vaginal prolapse (rectocele) may have symptoms of defecatory dysfunction. POP is most commonly classified using the POP Quantification system.^[4]^ Stage IV POP is defined as the protrusion of the distal portion to at least the total vaginal length (TVL) minus 2 cm beyond the hymen. As the POP stage increases, the incidence of vaginal bulging and urinary splinting becomes higher.^[5]^ Severe cystocele may eventually result in kinking of the urethra and could lead to voiding difficulty or paradoxical urinary continence.^[6]^

We report an unusual case of a 63-year-old married woman, who presented with a 3-year history of progressive increase in urinary frequency and involuntary urinary leakage, and was finally diagnosed with POP complicated with VVF.

## 2 Case presentation

A 63-year-old woman, para 3 (all spontaneous vaginal deliveries), with a medical history of cardiac arrhythmia for which she was taking beta-blockers, consulted our clinic. She complained of vaginal bulging sensation and involuntary urinary leakage for 3 years. The urinary leakage was continuous, had progressively worsened, and she even needed to wear diapers at night. Leading points of prolapse were upper anterior and posterior vaginal wall, point Ba (+7) and Bp (+8), respectively. Points Ap and Aa were both 2 cm distal to hymen (+2) and cervix (C) was totally everted and was 9 cm below the hymen (+9). The posterior fornix (D) was 6 cm below the hymen (+6). Cervix was elongated since point C was significantly more positive that point D. Measurements for perineal body, genital hiatus, and TVL were 2, 5, and 9 cm, respectively. Because TVL equals maximum protrusion, this is stage IV prolapse.

Besides, decubitus ulcer over cystocele with free leakage of urine from the ulceration was also observed. Therefore, the patient was admitted to our hospital under the diagnosis of POP complicated with VVF. We consulted a urologist for VVF repair and planned to perform pelvic floor reconstruction after VVF repair.

**Figure 1:**
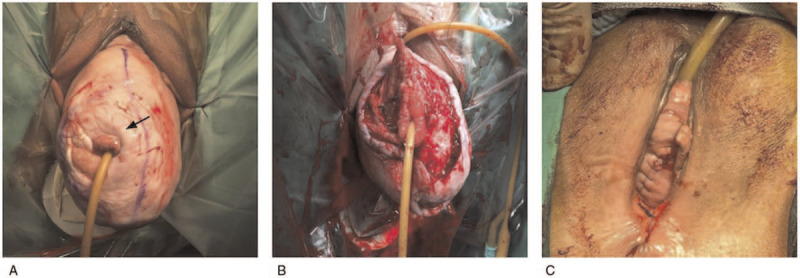
Gross picture of vesicovaginal fistula (VVF) and pelvic prolapse before and after repair. (A) A Foley catheter was inserted in the fistula (arrow). (B) The vaginal mucosa around the VVF was removed. (C) After the VVF repair, hysterectomy, and fixation, the pelvic prolapse was reduced.

During the operation, examination revealed that the VVF was located over the right posterolateral wall. A Foley catheter appropriate to the fistula size was inserted through the fistula into the bladder (Fig. 1A). When the balloon had been distended, the catheter was used to stabilize the bladder and expose the surgical field. A circumferential incision through the vaginal mucosa was made and the mucosa in this area was removed by sharp dissection (Fig. 1B). The fistula was closed in 2 layers. The tract of fistula was first closed with Vicryl 3 to 0. A second layer of interrupted sutures with Vicryl 2 to 0 was applied to close the prevesical fascia. After VVF repair, total vaginal hysterectomy (TVH) with sacrospinous ligament fixation (SSF) was performed. Anterior and posterior colporrhaphy was also performed after TVH and SSF (Fig. 1C). She was discharged home on postoperative day 4 with an in-dwelling Foley catheter for 2 weeks. Her postoperative course was uncomplicated and cystoscopy performed 14 days later revealed that the VVF repair site was healing well.

At one-year follow-up, pelvic examination showed that the vaginal wound had healed properly, and vagina was well-supported. The patient did not complain of vaginal bulging sensation and urinary leakage. The patient has provided informed consent for publication of the case.

## 3 Discussion

In addition to gynecologic surgery and obstructed labor, other causes of VVF include retroperitoneal, vascular or pelvic surgery, urologic or gynecologic instrumentation, infectious and inflammatory diseases, sexual trauma, vaginal laser procedures, external trauma, and vaginal foreign bodies.^[7]^ Vaginal fistulas can be caused by neglected vaginal pessaries for POP.^[8]^ However, our patient was not using pessaries. This report presents a case of VVF due to a rare cause that is, total uterine prolapse.

A previous report presented a case of grade III cystocele and VVF at the anterior vaginal wall. The patient underwent transabdominal repair for VVF with omental interposition and Brunch procedure for correcting the cystocele.^[9]^ Another study reported the case of a patient with vaginal prolapse for over 10 years. The patient did not use a pessary; however, cystoscopy revealed a large entrance of the VVF just inside the bladder neck. Transvaginal fistula repair with extensive anterior vaginal repair were performed.^[10]^ In light of the abovementioned findings, we can hypothesize that the POP produces an internal force which causes ischemia of the vaginal mucosa and pressure necrosis. Furthermore, this force probably leads to the breakdown of vaginal mucosa and development of a fistula.

There are many different methods for VVF repair. For small, non-malignant VVFs that are detected early, conservative management includes transurethral foley catheter placement with anticholinergic medication. Fibrin sealant or collagen can be used as an additional plug to help in closure of the defect. If conservative management fails, surgical intervention should be considered.^[7]^ The timing of repair depends on the condition of the surrounding tissue. Early repair might be carried out on healthy tissue, while surgery should be delayed up to 2 to 3 months to allow for recovery from inflammation, infection, or necrosis. VVF is most commonly repaired transvaginally, which has the merits of minimal blood loss, shorter hospital stay, and relatively less postoperative morbidity; and, at the same time, a success rate comparable with that of the abdominal approach.^[11]^ As for postoperative care, continuous bladder drainage through a Foley catheter for 2 to 3 weeks is suggested to avoid pressure on the suture line. In our case, VVF repair was performed through the vaginal route and was combined with pelvic organ reconstruction (TVH and SSF) to correct the POP. Patient was discharged home with an indwelling Foley catheter for 2 weeks.

In conclusion, VVF is a rare complication of POP. Our case illustrates that patients with POP should be carefully followed-up and the clinicians should be aware of the symptoms of VVF and the possibility of its development in severe POP. Early management of severe POP may prevent such complications.

## Author contributions

**Conceptualization:** Dah-Ching Ding.

**Data curation:** Ning-Shiuan Ting, Hsiang-Chen Lee, Jia-Ying Ke, Pei-Chen Li, Dah-Ching Ding.

**Formal analysis:** Ning-Shiuan Ting, Pei-Chen Li, Dah-Ching Ding.

**Supervision:** Dah-Ching Ding.

**Validation:** Dah-Ching Ding.

**Writing – original draft:** Ning-Shiuan Ting, Pei-Chen Li, Dah-Ching Ding.

**Writing – review & editing:** Dah-Ching Ding.
